# Luminescent Imaging of the Reverse Bias Behavior of Perovskite Solar Cells Using ReBEL

**DOI:** 10.1002/smsc.70329

**Published:** 2026-08-25

**Authors:** Jonathan Henzel, Klaas Bakker, Sjoerd Veenstra, Olindo Isabella, Luana Mazzarella, Arthur Weeber, Mirjam Theelen

**Affiliations:** ^1^ TNO Eindhoven The Netherlands; ^2^ Photovoltaic Materials and Devices Delft University of Technology Delft The Netherlands

**Keywords:** degradation, electroluminescence, imaging, partial shading, perovskite solar cells, reverse bias, stability

## Abstract

Partial shading remains a critical challenge for perovskite photovoltaics, as shaded cells in otherwise illuminated modules operate in reverse bias, which can accelerate degradation. In other solar cell technologies, reverse bias electroluminescence (ReBEL) imaging has been successfully employed to investigate the reverse bias breakdown mechanism. Here, ReBEL imaging is proposed as technique for imaging the local reverse bias current flow on perovskite solar cells. To that end, the ReBEL signal is affirmed to originate from radiative recombination in the absorber layer. ReBEL imaging is compared to established thermal current imaging techniques and reveals superior sensitivity and spatial resolution while showing similar features in the investigated samples. Further experiments reveal a dependence on the reverse bias current: The spatial distribution of the ReBEL signal differs significantly between small and large current injection levels. This could point towards two different breakdown mechanisms. These results show that, despite some open questions, ReBEL imaging could help understand the reverse bias behavior of perovskite solar cells better.

## Graphical abstract

Reverse bias electroluminescence (ReBEL) imaging provides a way to investigate the spatial distribution of the reverse bias current in perovskite solar cells. Our results show that the local reverse bias behavior depends strongly on the current injection level. At small currents, the cell edges and small spots break down. At large currents, the entire cell area passes current.

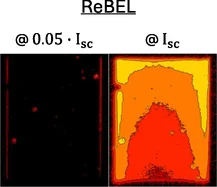

## 1 Introduction

The degradation caused by partial shading has been described as one of the major hurdles that are slowing down the introduction of perovskite photovoltaics (PV) into the market [1]. Such a partial shading event occurs when a part of a module area is shaded while the remaining module area is illuminated. Since modules typically consist of series‐connected cells, inhomogeneous photocurrent generation among the cells is undesirable.

In a simple partial shading scenario, only one of the cells is completely shaded and therefore, does not generate any current. However, this shaded cell still needs to pass the current generated by the illuminated cells. This requires it to operate at a negative voltage, i.e., the reverse bias (RB). In perovskite solar cells (PSCs), applying a reverse bias can quickly lead to dramatic degradation of the power conversion efficiency (PCE) [2, 3, 4].

Accordingly, significant research activity has been carried out on cell‐level, both in manipulating the breakdown voltage (a key metric for the design of modules) [5] and in understanding the breakdown and degradation mechanisms [6, 7, 8].

Research on (mini‐)modules is also available: Aninat et al. performed partial shading experiments on perovskite modules and observed pronounced degradation restricted to parts of the shaded cell [9]. Their interpretation attributes this effect to spatial variations in the breakdown voltage, leading to locally increased reverse bias current densities. These elevated current densities, in turn, induced increased degradation. Their findings demonstrate the importance of uniform reverse bias breakdown for partial shading stability. Nevertheless, to the best of our knowledge, a detailed investigation of the spatial (in)homogeneity of the reverse bias behavior of cells has not yet been reported.

In many studies on cell‐level, perovskite solar cells are assumed to be spatially homogeneous or 1D in regards to the reverse bias behavior. Thermography‐based imaging techniques are occasionally used but typically only to show the presence of shunts. Examples are the publications by Bowring et al., Najafi et al., Bogachuk et al., and Li et al. who used steady‐state thermography (ssIR) [3, 10, 11, 12]. In the work of Razera et al., the authors employed reverse bias dark lock‐in thermography (RB‐DLIT) [2].

In other solar cell technologies (i.e., silicon‐ and CIGS‐based), reverse bias electroluminescence (ReBEL) imaging is a technique that has been used to great effect to investigate the local reverse bias behavior [13, 14].

The goal of this publication is to investigate the application of ReBEL imaging as a tool for investigating the local reverse bias behavior of perovskite solar cells. To that end, we show that PSCs exhibit electroluminescence when reverse biases are applied. We compare ReBEL to RB‐DLIT and ssIR imaging. Finally, the relationship between local ReBEL emission rate and applied reverse bias is examined. The presence of two different regimes at different current injection levels is revealed. These results shows that ReBEL imaging could be used as a supplement or alternative to thermal imaging techniques.

## 2 Experimental

**FIGURE 1:**
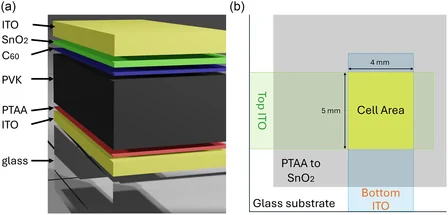
A cross‐section of the perovskite solar cells (a) and a top‐view of the cell design (b).

In this work, we study semitransparent perovskite solar cells in a p‐i‐n configuration. The cell area is $0.2\;{\mathrm{cm}}^{2}$ and the perovskite is a triple cation, double halide formulation (${\mathrm{Cs}}_{0.05}{\mathrm{MA}}_{0.15}{\mathrm{FA}}_{0.8}{\mathrm{PbI}}_{2.7}{\mathrm{Br}}_{0.3}$) with a band gap of around 1.6 eV. The complete layer stack and the solar cell design are shown in Figure 1a,b. On each glass substrate, four isolated solar cells are fabricated that are rotated by 90% to each other. The details of the fabrication process are described in the Note S1.

The results of the initial characterization, consisting of JV measurements and maximum‐power‐point‐tracking (MPPT), are presented in the Note S2. The median efficiency of the batch of 56 cells was 15.5% for the forward sweep, respectively 15.3% for the reverse sweep. This is caused by a low fill factor (median 70.8%, 69.6%) due to large resistive losses in the top electrode. We forwent metal electrodes or even metal fingers that could have decreased these losses since they disturb the imaging techniques that are the focus of this publication. Additionally, the presence of metal electrodes have been convincingly related to shunting under reverse bias.

Before proceeding with describing the experiments, we clarify our nomenclature.

The current (I) measured under illumination is negative. The approximate short‐circuit current of our cells is $I_{\mathrm{sc}}$ = −4 mA; the corresponding current density is $J_{\mathrm{sc}}=-20\;\mathrm{mA}\;{\mathrm{cm}}^{-2}$. Under reverse bias, the injected current flows in the same direction, and so is negative as well (e.g., I = $I_{\mathrm{sc}}$ = −4 mA). In contrast, the current that is injected during (forward) electroluminescence measurements is positive, e.g., I = $-I_{\mathrm{sc}}$ = 4 mA.

**FIGURE 2:**
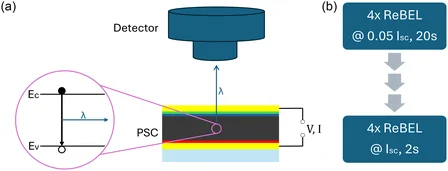
The basic principle of EL and ReBEL measurements (a) and the experimental procedure of the ReBEL sweep (b).

The basic principle of EL and ReBEL measurements is displayed in Figure 2a. For spectrally dependent EL and ReBEL measurements, we use a Renishaw Raman system to make use of its microscope and its cooled camera. The sample is excited using a Keithley source/measure unit as current source. We measure from 630  to 850 nm. For the EL measurement (I = 4 mA), we accumulate signals for 10 s. For the ReBEL spectrum (I = −4 mA), we integrate over 100 s in total. The software Wire 3.4 by Renishaw is used to control the system.

**FIGURE 3:**
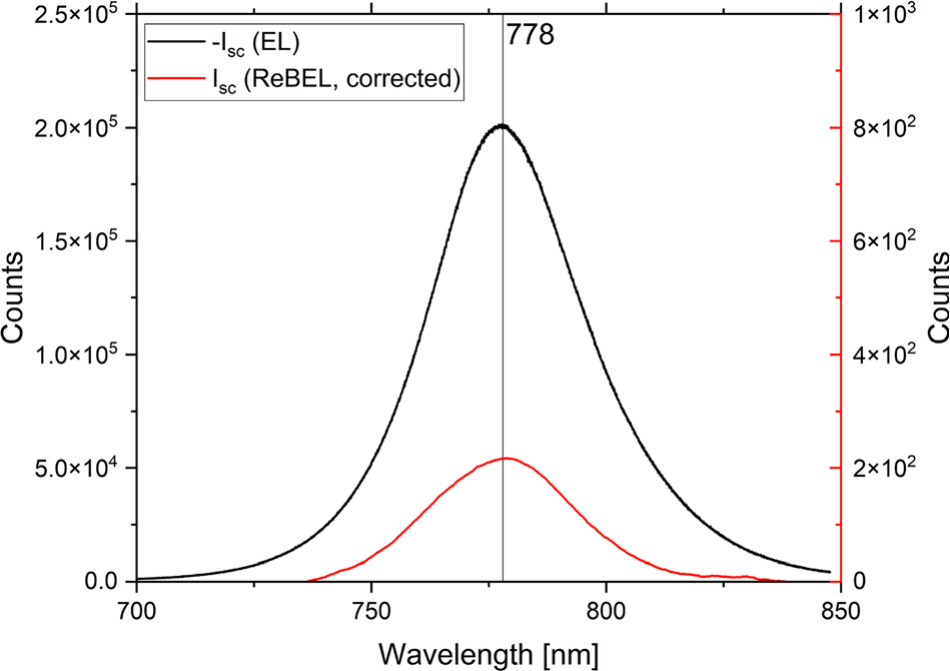
The emission spectra of EL ($-I_{\mathrm{sc}}$) in black and ReBEL ($I_{\mathrm{sc}}$) in red. The ReBEL spectrum has been smoothed and baseline‐corrected.

To acquire a ReBEL image, we inject a reverse bias current (I) for a time (t) while a camera (greateyes LumiSolarCell system with a 16‐bit Si‐CCD) detects light emission from the “n‐side” of the sample. The local light emission is integrated over t and depicted as an image. Before and after this integration time, the current source (Keithley 2400) is switched off. The reverse bias that is applied to the sample during the integration time is logged with a frequency of approximately 100 Hz.

The core experiment of this publication is the ReBEL sweep. Its procedure and important parameters are shown in Figure 2b and in Table 1.

**TABLE 1:** Important measurement settings of a ReBEL sweep.

I/$I_{\mathrm{sc}}$	0.05	0.1	0.15	0.25	0.375	0.5	0.625	0.75	0.875	1
I [mA]	−0.2	−0.4	−0.6	−1	−1.5	−2	−2.5	−3	−3.5	−4
J [mAcm 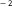 ]	−1	−2	−3	−5	−7.5	−10	−12.5	−15	−17.5	−20
t [s]	20	10	8	4	3	2	2	2	2	2

We acquire a sequence of ReBEL images, starting at small RB currents and proceed towards larger RB currents. During this sweep, t is regularly adjusted to provide a signal strength above the camera noise level while not overloading the CCD. In order to account for the meta‐stability of PSCs under reverse bias, we repeat the image‐taking at every I‐level four times. That provides us with information about the changes caused by the reverse bias during the measurement and allows the sample to reach a quasi steady‐state. This is shown in the Note S3, where it is visible that the average applied voltage and the emission rate in the general cell area converge to limits with successive measurements. At most current levels, the changes after three measurements are minimal. Therefore, the fourth images taken at each current level are shown in the following analysis. A more detailed discussion can be found in the Note S3. The full data set, so the ReBEL images 1–4, is available at a data repository (see Data Availability).

For analyzing the resulting ReBEL images, we proceed as follows: as the voltage is not constant when a fixed reverse bias current is injected, we calculate a mean voltage from the voltages logged during the recording of a ReBEL image. This is shown in Note S4. The emission intensity is divided by the integration time to receive a emission rate ER. To gain information about the local behavior, small parts of the observed area are chosen and a mean local ER calculated: $ER_{\mathrm{avg}}$. In the ReBEL images depicted below, these areas are marked with blue rectangles.

Additionally, we imitate the ReBEL‐sweep with two other imaging techniques.

Firstly, we perform ssIR thermography. We inject currents for a certain time and record images with a frequency of 5 Hz, using a InSb camera of InfraTec. The time set at a constant current is consistent with the ReBEL integration times at the corresponding current level. The local heating is displayed after subtracting a baseline‐image that was acquired before the current injection started.

Secondly, we employ RB‐DLIT. There, we apply *voltages*, starting at small reverse biases and proceeding towards larger reverse biases. For every applied bias, we take an image with a measurement duration of 30 s, a pulse‐duty factor of 0.5, a frame rate of 100 Hz, 200 frames per second, and a Lock‐In Frequency of 0.5 Hz. DLIT being a lock‐in technique, the voltage is switched on and off with the Lock‐In Frequency to create a wave‐like disturbance. By filtering out the parts of the measured signal that do not follow this waveform and averaging over many images, the spatial resolution can be improved. The same camera as for the ssIR thermography is used.

## 3 Introducing ReBEL as Imaging Technique

### 3.1 Do Perovskite Solar Cells Exhibit ReBEL Behavior?

The most basic requirement for ReBEL imaging is that perovskite solar cells show electroluminescence when reverse biases are applied. To test that, we performed spectrally resolved measurements at $-I_{\mathrm{sc}}$ (EL) and at $I_{\mathrm{sc}}$ (ReBEL) (Figure 3).

The EL spectrum shows a strong signal with a peak centered at λ≈778 nm. The ReBEL spectrum is depicted after smoothing and a baseline‐correction. The original data can be found in the Note S5. It shows a peak centered at the same wavelength as the EL spectrum (λ≈778 nm). This wavelength corresponds to the band gap of the perovskite of $E_{g}\approx 1.6\;\mathrm{eV}$.

While the wavelength‐dependence of EL and ReBEL is alike, the intensity of emission shows a difference of several orders of magnitude. This is likely related to the direction of the current. In both situations, the same (absolute) current is flowing through the device. However, in EL, the diode is operating in the ‘right’ direction while the diode is forced into breaking down in the ‘wrong’ direction in ReBEL. It is not surprising that less radiative recombination occurs in the ReBEL case.

These results confirm that perovskite solar cells emit photons when operating in reverse bias. As the peak corresponds to the band gap, the emission seems to occur in the perovskite layer with radiative recombination via the band gap. This matches the observations of Puttnins et al. for CIGS solar cells [14]. They also found that the spectra of EL and ReBEL coincide. They explained that phenomenon with a second diode that is inversely, serially connected to the “main diode.” In contrast, the spectrum for ReBEL in microcrystalline silicon solar cells is more complex and the result of several mechanisms occurring in parallel [15, 16].

To the best of our knowledge, this is the first time that the ReBEL spectrum of a perovskite solar cells is reported. The detailed mechanism of photon emission underlying ReBEL (beyond it being radiative recombination in the perovskite layer) remains material for future research. However, the similarity to the EL spectrum could be indicative of a similar mechanism occurring in reverse bias and of similar laws being applicable.

### 3.2 Can ReBEL Imaging Be Used to Characterize the Reverse Bias Behavior?

After having shown that the perovskite solar cells used in this study emit light when reverse biased, the next step consists of testing whether we can use this photon emission to image the local reverse bias behavior. To that end, we compare images gained from ReBEL to images gained via more established thermal imaging techniques: steady‐state infrared thermography (ssIR) and reverse bias lock‐in thermography (RB‐DLIT).

These thermal imaging methods map the surface temperature distribution of the sample. There are various mechanisms leading to heat generation in solar cells in the dark, e.g., Joule heating and thermalization of (injected) hot carriers [17]. Generally, the locally dissipated power is measured which depends on the local voltage and local current density.

ssIR imaging can be performed at a constant current; however, it often suffers from the effects of lateral heat transport. This leads to a “smearing” of the signal and reduced resolution and sensitivity [17]. Using lock‐in techniques (e.g., RB‐DLIT) is a way of improving both by applying a periodic excitation (e.g., a current). The lock‐in principle reduces noise and increases resolution [17]. However, as perovskite solar cells react only slowly to changing electrical conditions, RB‐DLIT images with high lock‐in frequencies might not be representative of the behavior at steady‐state. Therefore, relatively large lock‐in frequencies are employed in this experiment, which limits the advantages usually resulting from a lock‐in imaging technique.

**FIGURE 4:**
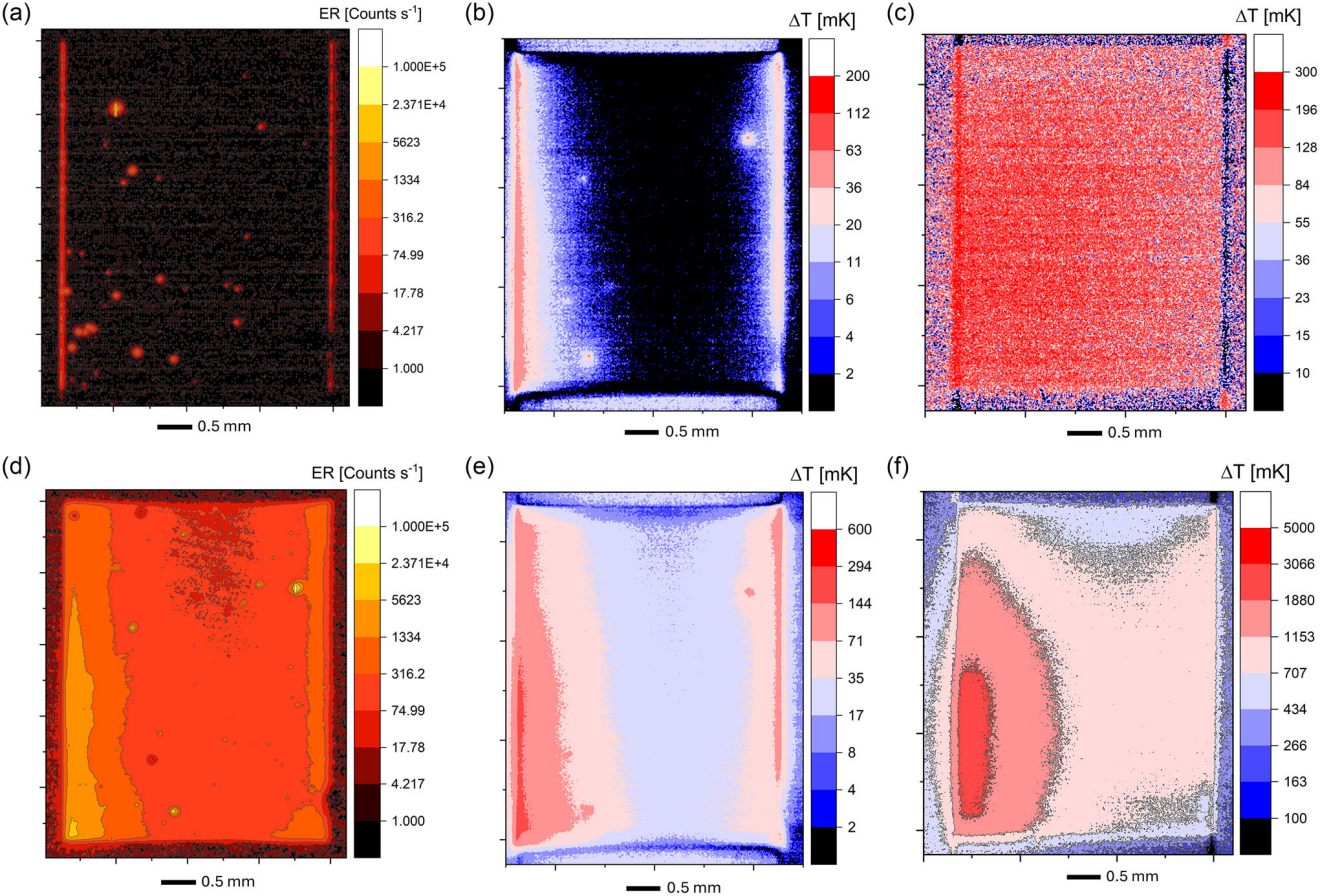
ReBEL images at $0.05\;I_{\mathrm{sc}}$ (a) and at $I_{\mathrm{sc}}$ (d), respectively. RB‐DLIT images at −5.5 V (b) and at −6 V (e), and ssIR images at $0.05\;I_{\mathrm{sc}}$ (c) and at $I_{\mathrm{sc}}$ (f). ReBEL and RB‐DLIT show largely the same features while ssIR only agrees with the other techniques at a large current. However, the details are clearest in the ReBEL images, and the least visible in ssIR.

We recorded several ReBEL images at various reverse bias current levels, then several RB‐DLIT images at various reverse biases, and finally several ssIR images at various reverse bias current levels on the same sample. On each current or voltage level, only one image was taken (contrasting the ReBEL sweep that is described in 2 Experimental). We started with small reverse bias currents and reverse biases and proceeded towards larger values. Results of this experiment are presented in Figure 4. Similar results from other samples can be found in the below‐mentioned data repository (see Data Availability).

The top row (Figure 4a–c) shows contour plots from all three imaging techniques at a small RB current. In this state, only a small reverse bias current is passing through the cell. The RB‐DLIT image at this voltage (−5.5 V) was chosen due to its similarity to the ReBEL image as we could not measure the current during the RB‐DLIT measurement.

The bottom row (Figure 4d–f) shows the corresponding images at a large RB current. In this state, a substantial current is going through the cell and it operates past its breakdown. In the case of ReBEL and ssIR at $I_{\mathrm{sc}}$, in the case of RB‐DLIT at −6 V.

In all images, the emission intensity (for ReBEL) or the temperature increase (ΔT) (for ssIR, RB‐DLIT) are depicted in logarithmic color scales. The x‐ and *y*‐axes represent the spatial extent of the samples. Since the camera used for the ReBEL‐sweep is a different one than for ssIR and RB‐DLIT, the resolutions are different. Therefore, the number of pixels that measure the width and height of the cell are different, as well. The images were scaled to approximate each other in size.

We start by describing the images at a small RB current (Figure 4a–c). The ReBEL image (Figure 4a) shows a cell area that is largely dark. Only bright vertical stripes at the edge of the cell area and a multitude of small bright spots are visible. The corresponding RB‐DLIT image (Figure 4b) shows warm vertical edges. Especially the left edge and the bottom corner are bright and extend into the cell area. Additionally, there are a few warm spots visible in the cell area. Finally, the ssIR image (Figure 4c) shows a very slight heating in the whole cell area. Additionally, the left edge of the cell seems warmer than the middle part of the cell area and the right edge colder.

When comparing these images, some observations stand out: The ReBEL image and the RB‐DLIT image agree relatively well with each other. Both show bright edges, a cell area that is not very active, as well as some bright spots in that area. The ReBEL image shows far more bright spots which can partially be assigned to bright spots in the RB‐DLIT image. Additionally, the distinction between edge and cell area is clearer in the ReBEL image than in RB‐DLIT. In contrast, the ssIR image just shows a slight uniform heating up of the whole cell area. The warmer and colder edges are a measurement artifact as is shown in Note S6.

Proceeding to the images at large RB currents (Figure 4d–f), we see in the ReBEL image (d) that large parts of the cell area are lighting up, even if most of the intensity comes from the regions near the edges and the bottom left corner. Some bright spots in the cell area are still present. They do not seem to have an impact on the surrounding area. The corresponding RB‐DLIT image (Figure 4e) shows a strong signal along the vertical edges on the left and right of the cell area. Especially the left edge and the cell area near it are bright. Additionally, some bright spots are visible. In the ssIR image (Figure 4f), we see the bottom left corner heating up strongly which radiates into the surrounding area, even outside of the cell. Furthermore, the remaining cell area seems to heat up relatively homogeneously. Only the bottom right corner and the top center are slightly cooler. Generally, the area outside of the cell area appears to have heated up on all sides of the cell.

When comparing the techniques at large RB current, we make the following observations: All three techniques show that the cell area is now active as well, even if only at a low level. They also all show that the bottom left corner is especially active. Additionally, in ReBEL and RB‐DLIT, the top center is clearly less active than the bottom center. This is less obvious in the ssIR image. The edges are not visible anymore in ReBEL but are still present in RB‐DLIT. In ssIR, they have not been visible even at small RB currents and are still not present. The remaining bright spots that are visible in ReBEL and RB‐DLIT correspond to each other according to their location. However, there are more and weaker bright spots visible in ReBEL, while ssIR does not show any bright spots.

To summarize our observations, the ReBEL and RB‐DLIT images show largely similar features. The slight differences that are present could be explained by the current‐defined state of the ReBEL images and the voltage‐defined state of the RB‐DLIT image not corresponding to each other perfectly. The presence of more bright spots in the ReBEL images might be explained, in part, by a higher sensitivity and resolution. Also, changes to the sample due to the ReBEL measurements could have occurred that altered the local behavior at bright spots in the RB‐DLIT measurements.

On the other hand, the ssIR images show far less details that could be compared to the images resulting from the other techniques. At least, at a large reverse bias current, the warming in the bottom left corner corresponds to similarly increased activity in the ReBEL and the RB‐DLIT images. That could imply that the sensitivity and resolution of the technique is simply to low to display the features that can be observed in the other images.

Our conclusion of the results of this comparison of imaging techniques is that ReBEL imaging in this current/voltage regime seems to reveal the same qualitative information as thermal imaging (especially RB‐DLIT) via a different mechanism. That means, from ReBEL imaging, we can at least draw tentative and qualitative conclusions about the local reverse bias current densities and reverse bias (breakdown) behavior. At the same time, ReBEL imaging shows advantages in sensitivity and resolution. However, for more reliable quantitative results, e.g., local JV curves, the mechanism of ReBEL needs to be clarified in more detail.

## 4 Using ReBEL to Investigate the Local RB Behavior

After having presented results that imply that ReBEL can be used to image the reverse bias behavior of perovskite solar cells, the next step is to show, using our cells as an example, what ReBEL imaging can reveal. To that end, we have performed the ReBEL sweep experiment (described in 2 Experimental) on our solar cells and discuss the results in detail in the following.

### 4.1 Qualitative Analysis

**FIGURE 5:**
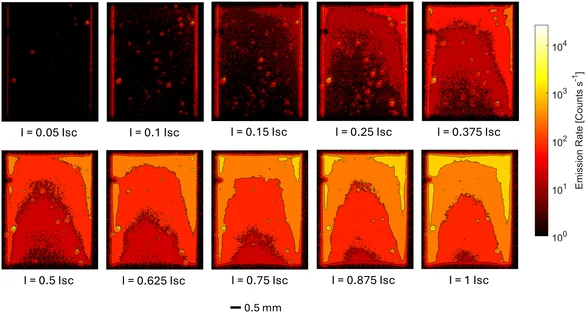
The results of a ReBEL sweep experiment. For each current injection level, the fourth image is shown. The emission rate is displayed on a logarithmic color scale.

In Figure 5, the results of a ReBEL sweep are presented. For each current injection level, the fourth ReBEL image is shown as it is the most stabilized (see 2 Experimental). The emission rate is displayed on a logarithmic color scale. Similar results from other cells and the complete data (e.g., the images 1–3 for each current injection level) can be found in the data repository that is described in Data Availability.

At the smallest injection current ($I=0.05\;I_{\mathrm{sc}}$), the only visible intensity is emitted from the two thin stripes at the side edges of the cell area and a few bright spots in the cell area. The general area of the cell is dark.

When the reverse bias current is increased, the edges increase in brightness and more bright spots appear at first. However, at a certain current ($I=0.15\;I_{\mathrm{sc}}$), the cell area itself begins lighting up as well, in parallel and apparently independent of the bright spots. This begins in the top right corner.

Already at $I=0.375\;I_{\mathrm{sc}}$, the whole cell area is active. At the same time, many bright spots are still distinct from the surrounding area. In contrast, the edges of the cell are barely distinguishable from the cell area anymore.

With larger reverse bias currents, the cell area gets brighter. A gradient of the emission rate remains clearly visible. Clearly, the ReBEL activity is higher at the top of the cell than at the bottom. It is possibly also slightly higher on the right side than on the left side. Finally, the cell area near the side edges also is brighter than the center of the cell area.

At the same time, most bright spots do not significantly increase in brightness and are therefore ”swallowed” by the surrounding area when it reaches a similar order of magnitude of the emission rate. Some especially bright spots, however, remain visible even at the largest injected current level. The edges are not visible at all anymore at larger reverse bias currents. Their former position is taken up by a liminal area between the bright cell area and the dark dead area outside of it.

These observations agree with those shown in Section 3.2 where we compared the imaging techniques. It should be noted that the orientation of the cell here is rotated by 180%.

These results reveal a significant difference between the appearance at small currents and large currents in our cells. At small reverse bias currents, the cell edges and bright spots are mainly responsible for the ReBEL emission. The general cell area is largely inactive. On the other hand, at large currents, the cell area dominates the ReBEL emission, even if it is spatially not homogeneous. Here, the cell edges and most of the bright spots are not distinguishable from the cell area anymore. If we interpret ReBEL emission as a sign of local reverse bias current flow (as discussed in Section 3.2), this difference could indicate the presence of two different reverse bias breakdown mechanisms in our solar cells.

### 4.2 Semiquantitative Analysis

**FIGURE 6:**
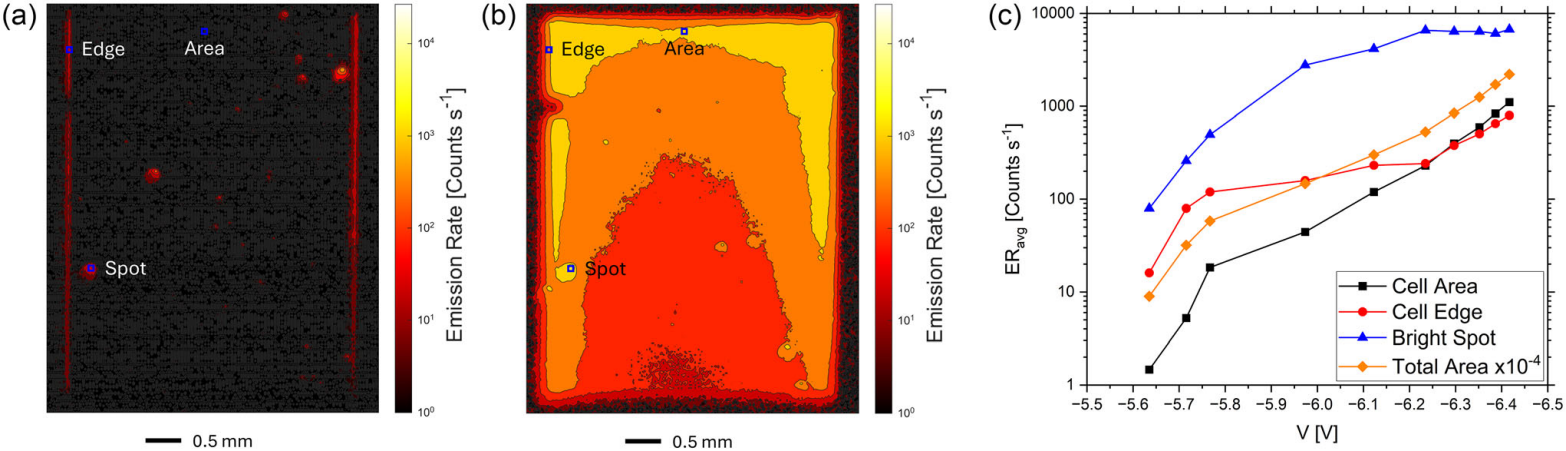
The areas that were used as examples for the semiquantitative analysis are marked as blue squares in a small (a) and large (b) current ReBEL image. The average emission rates of these areas and the average total emission rate are plotted semilogarithmically (c). The $ER_{\mathrm{avg}}$ of the total area is multiplied by $10^{-4}$ to make the comparison easier.

The ReBEL sweep experiment allows the extraction of semiquantitative information by relating the local emission rate to the applied reverse bias. This is shown in Figure 6 by examples for the cell area, the cell edges, and a bright spot. Their average emission rates are displayed semilogarithmically against the reverse bias. Other examples for the cell area, the edges, and bright spots can be found in the Note S7. In the graph in Figure 6c, the emission rate of the total area is used as comparison. To ease that, its average emission rate is multiplied by the factor $10^{-4}$.

It is clearly visible that different relationships between emission rate and applied reverse bias are present. The bright spot shows the highest emission rates, yet seems to reach a saturation at large reverse biases. The average emission rate at the cell edges increases strongly at small reverse biases but reaches a point of saturation at V = −5.8 V. At large reverse biases, its emission rate seems to increase again. However, that is an artifact as the edge is not distinguishable from the cell area anymore at these reverse biases.

The part of the cell area used as example here shows very small emission rates at small reverse biases as was already visible in Figure 5. From the third data point at V = −5.8 V, the behavior changes suddenly, corresponding to the cell area beginning to light up in the third image of Figure 5. At larger reverse biases, a nearly linear increase of the average emission rate is visible, corresponding to an exponential relationship with the reverse bias. In general, the behavior of the total area is most closely mirrored by the chosen part of the cell area. That indicates that, while bright spots are present even at large currents, the behavior of the cell seems to be dominated by the cell area.

Both the spatial distribution of the ReBEL emission rate in Figure 5 and the semiquantitative analysis of the local ReBEL emission rate point to the same conclusion: Significant differences in the behavior separate the cell edges (and bright spots) from the cell area. This points either at two different mechanisms of ReBEL emission or at two different breakdown mechanisms. However, as the thermal imaging techniques showed similar features, we tentatively conclude that, in our solar cells, two different breakdown mechanism are occurring at different current injection levels.

Two other observations requiring additional attention are the bright edges of the cells at small current levels and the inhomogeneity of the emission rate at large current levels. These will be discussed in the following sections. The bright spots are more difficult to pin down as they do not show one behavior (see Note S7). They will have to be the focus of a future publication.

### 4.3 Bright Edges

The feature that we call "bright edge" is only clearly visible at small reverse bias currents ($I\leq 0.375\;I_{\mathrm{sc}}$). It describes two vertical stripes at the left and right edges of the cell area with increased ReBEL emission rates. At larger reverse bias currents, they are not distinguishable anymore from the brighter cell area.

Their location is the key information for explaining their origin. The orientation of the cell in Figures 5 and 6 corresponds to the schematic of the cell design that is displayed in Figure 1. That means that the location of the bright edges match with the edges of the bottom ITO electrode.

The bottom ITO electrode has a thickness of ca. 150 nm. Its overlap with the top electrode defines the cell area. It is fabricated by full‐area sputtering, followed by a photolithography process to pattern the layer. On this scale, photolithography leads to a step‐like edge as is shown by stylus profiling results in the Note S8a.

**FIGURE 7:**
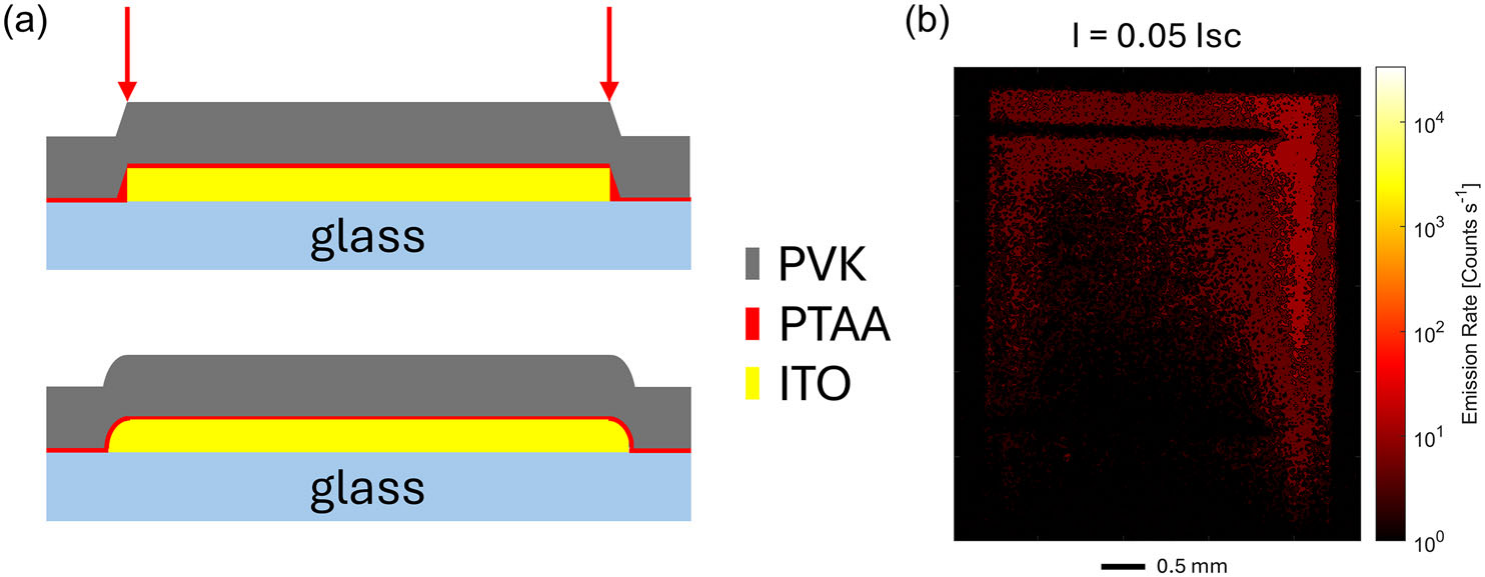
Schematics showing the step‐like edges (top) and the rounded edges (bottom) of the bottom ITO electrode with the glass substrate, hole transport layer PTAA, and the perovskite layer (a). A ReBEL image of a solar cell with rounded bottom ITO edges at a small reverse bias current (b).

This situation is displayed in Figure 7a in the top schematic. To test whether the step‐like step geometry of the ITO electrode really is responsible for the observed bright edges, we fabricated solar cells with more rounded bottom ITO electrodes. This is displayed in Figure 7a in the bottom schematic. The relevant profiling results can be found in the Note S8b.

A resultant ReBEL image, taken at a small reverse bias current $I=0.05\;I_{\mathrm{sc}}$ is shown in Figure 7b. There, no bright edges are visible. Instead, parts of the cell area are already showing ReBEL emission. The black horizontal stripe is part of a metal electrode on top of the top ITO electrode. This confirms that the geometry of the bottom ITO is responsible for occurrence of the bright edges.

For the mechanism of how the step‐like ITO edge is connected to locally increased ReBEL emission, several different explanations are possible.

The layer on top of the ITO electrode is the hole transport layer PTAA which has an approximate thickness of only 10 nm. At these step‐like edges, this could lead to insufficient coverage or, at least, a locally very thin PTAA layer. Insufficient coverage of the ITO electrode by a hole transport layer has been connected to small breakdown voltages on cell level in a recent publication [5]. Here, a local breakdown voltage that is smaller than the breakdown voltage of the general area could be present and explain the increased ReBEL emission rates at smaller reverse biases than the cell area.

Other explanations could involve the formation of non‐ohmic shunts at the edges due to direct contact between electrode and perovskite. Another alternative might be a local increase of the electric field strength due to the sharp geometry of the ITO electrode. However, ascertaining the exact mechanism is the work of a future publication.

### 4.4 Cell Area

The inhomogeneity of the ReBEL emission in the cell area is the second observation that we want to discuss in more detail. In Figure 5, the cell area begins lighting up at $I=0.15\;I_{\mathrm{sc}}$ in the top right corner. With increasing reverse bias current, additional parts of the cell area also begin emitting ReBEL, seemingly spreading from the top to the bottom. This vertical gradient (small emission rates at the bottom and large emission rates at the top) remains present at all larger current injection levels. Additionally, a smaller horizontal gradient (larger emission rates to the right than to the left) might be visible additionally. Furthermore, it seems that the cell area near the cell edges to the sides is more active than the center of the cell area. At the largest reverse bias current ($I=I_{\mathrm{sc}}$), a difference of several orders of magnitude of the emission rate is visible between different parts of the cell area.

Previously, we argued that ReBEL likely follows similar laws as (forward) EL. This was based on the observation of the same emission spectrum (Figure 3). The near‐linear relationship of the logarithm of the emission rate with the reverse bias at large reverse biases (Figure 6c) also fits the exponential dependence described for EL [18]. Therefore, we compare the previously presented results of ReBEL imaging with results of EL imaging.

**FIGURE 8:**
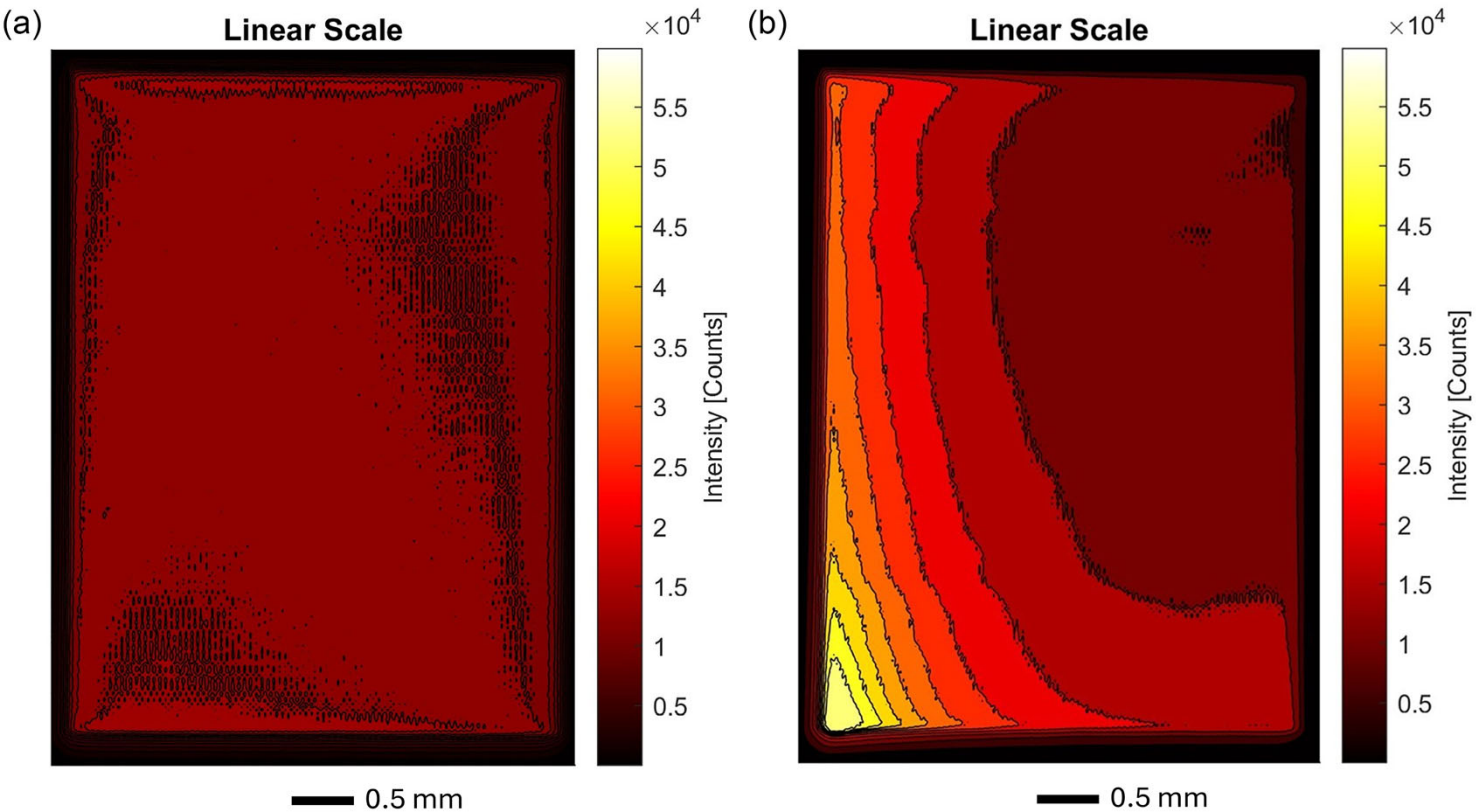
Results of EL imaging measurements at a small current $I=-0.1I_{\mathrm{sc}}$ (a) and a large current $I=-I_{\mathrm{sc}}$ (b). The emission intensity is shown on a linear color scale. The orientation of the cell agrees with the orientation of the ReBEL images in Figure 5 and the schematic cell design in Figure 1b.

The results of EL imaging at a small current ($I=-0.1\;I_{\mathrm{sc}}$) and a large current ($I=-I_{\mathrm{sc}}$) are displayed in Figure 8. The EL intensity is shown on a linear color scale. The measurement was performed before the ReBEL sweep on the same cell as the ReBEL sweep of Figure 5. Therefore, the cell is oriented (like in the ReBEL images) as displayed in Figure 1b.

According to the laws governing EL (as described in Haunschild et al. [18]), the electroluminescence intensity is highly sensitive to differences of the local series resistance. Due to the exponential dependence of the intensity on the local diode voltage, the impact of the series resistance is especially visible at large current injection levels. It stands to reason that the intensity gradients visible in Figure 8b are caused by the series resistance of the electrodes.

Two separate intensity gradients are visible: one very steep gradient is oriented vertically from the top to the bottom of the cell. Another, slightly less steep gradient is oriented orthogonally from the right to the left of the cell. These gradients match well with the orientation of the bottom and the top ITO electrodes, respectively.

A comparison with the ReBEL images in Figure 5 at the corresponding absolute current injection levels reveals certain similarities and differences: The vertical gradient of the emission rate is clearly visible in ReBEL as well. The horizontal gradient is less clearly present at the largest current level but might be present at smaller injection levels. However, the gradients in the ReBEL images are inverted in relation to the gradients visible in EL. In EL, the intensity decreases from the bottom to the top; in ReBEL, the emission rate increases dramatically in the same direction. Finally, in the ReBEL images, the center of the cell area shows smaller emission rates than the edges to the sides of the cell area. This observation does not have a parallel in the EL images.

At this point, we do not have an explanation for the inverted vertical and horizontal gradients of the emission rate in ReBEL. To outline possible future directions of research, we will at least provide some speculation.

It is a possibility that these gradients are connected to the electrodes as their orientation fits very well. However, as per our understanding, the direction of the current should not influence the impact of the series resistance of the ITO electrodes. That is a question that we have to leave open for a future investigation.

The brighter side edges of the cell area could be connected to the same bottom ITO geometry as the "bright edges" at small current injection levels. Then, this effect would be stretched on both sides towards the center of the cell area. An argument against this is the observation in Figure 6b where the blue square, marking the position of the bright edge, can be seen to be just outside of the yellow area with the largest emission rate. Therefore, the increased emission rate at small currents and the increased emission rate at large currents at the edge are present at slightly different locations. That could mean that two different mechanisms occur near the edge: The first occurs just outside the cell area at small currents, while the second one occurs just inside the cell area at large currents.

Alternatively, lateral ion migration at the borders of the cell area as shown in the publication by Jacobs et al. could serve as partial explanation [19]. Lateral ion migration would increase the concentration of mobile ions near the borders of the cell area and therefore decrease the local breakdown voltage, leading to increased local ReBEL emission rates. The main argument against this hypothesis is that it does not explain why we do not see increased local emission rates near the bottom edge of the cell area.

In this section, we have qualitatively and semiquantitatively analyzed the results of a ReBEL sweep on our perovskite solar cells. We observed that the bright edges and bright spots dominate the ReBEL emission at small reverse bias currents, while the general cell area dominates at large reverse bias currents. Investigating the cell area, the cell edges and a bright spot semiquantitatively, we found that these features differ in their relationship between the local emission rate and the reverse bias. We concluded tentatively that we observe either two different mechanisms of ReBEL emission or of reverse bias breakdown.

More detailed investigations of the bright edges revealed that they are likely connected to the step‐like edges of the bottom ITO electrode. When these sharp electrodes were exchanged for more rounded electrodes, the bright edges are not present anymore in ReBEL imaging. The exact mechanism, e.g., local non‐ohmic shunts or a locally smaller breakdown voltage, remains unclear, however.

Finally, the inhomogeneity of the emission rate in the cell area at large reverse bias currents was discussed. A comparison with EL imaging showed that the observed spatial inhomogeneity could be connected to the series resistance of the electrodes seem to be present in ReBEL, however inverted. That hypothesis would additionally require another effect to to increase the emission rates at the edges of the cell area. Possible explanations involving lateral ion migration and an effect of the geometry of the bottom ITO electrode were discussed shortly. Both for explanations of the inversion of the effects of series resistance and of the effect of the edges at large currents, we have to refer to future research.

## 5 Conclusions

The aim of this study was to test whether ReBEL imaging could be used to investigate the reverse bias behavior of perovskite solar cells. To that end, we showed that the dominating ReBEL signal stems from radiative recombination in the perovskite layer, based on the spectrum of the recorded ReBEL emission. However, due to the low intensity of the emission and limits to the measured wavelength range, we can not categorically exclude the possibility of other weaker emission peaks related to other mechanisms of photon emission. The comparison with thermal imaging techniques (especially the strong similarity of ReBEL and RB‐DLIT) led us to the conclusion that the ReBEL signal is at least qualitatively related to the local reverse bias current density. However, the quantitative relation between local current density and ReBEL emission rate is unknown. This significant limitation stems, firstly, from the lack of surety about the ReBEL mechanism: while in forward EL, the series resistance generally dominates the spatial inhomogeneity at large currents, our imaging results leave that question open. They point towards either other effects playing a more important role or towards a as‐of‐yet unexplained inversion of the effect of the series resistance. The second reason for the lack of surety arises from the uncertainty about the reverse bias breakdown mechanism. The breakdown and therefore the local current density might be governed by parameters that are unique to the reverse bias, e.g., local density of mobile ions or the local thickness of a transport layer. As the influence of these factors and their spatial inhomogeneity are unclear, assumptions about the spatial distribution about the local current density are difficult to make.

Thus, our results show that the imaging of the reverse bias behavior of perovskite via ReBEL is possible and that it promises advantages over thermal imaging techniques. However, a more detailed understanding of its correct interpretation and the factors influencing it is required. Especially the influence of the cell architecture on the spatial inhomogeneity of the ReBEL signal requires a more thorough investigation to disentangle behavior specific to this cell design from general reverse bias behavior. Performing the same experiments on samples with a significantly different design would be an easy way of investigating this question.

## Author Contributions

**Jonathan Henzel:** methodology, formal analysis, resources, investigation, writing – original draft, visualization. **Klaas Bakker:** methodology, resources, writing – review & editing. **Sjoerd Veenstra:** project administration, writing – review & editing. **Olindo Isabella:** supervision, writing – review & editing. **Luana Mazzarella:** supervision, writing – review & editing. **Arthur Weeber:** supervision, writing – review & editing. **Mirjam Theelen:** funding acquisition, supervision, writing – review & editing, project administration.

## Funding

This study was supported by Ministry of Climate Policy and Green Growth and Ministry of Economic Affairs.

## Conflicts of Interest

The authors declare no conflicts of interest.

## Supporting information

Supplementary Material

## Data Availability

Additional data is available in the Supporting Information. The full data set for this article is available at 4TU.ResearchData at https://doi.org/10.4121/a6e75aaa‐8051‐4925‐9ee7‐3c3beb2fc569.
