# Supplementary material for: Luminescent Imaging of the Reverse Bias Behavior of Perovskite Solar Cells Using ReBEL
Source: Small Sci. 2026 Aug 25;6(8):e70329. doi: 10.1002/smsc.70329 (PMC13516957; doi:10.1002/smsc.70329)
Supplement: Supplementary file 1 [file SMSC-6-e70329-s001.pdf]

Electronic Supporting Information for: ReBEL - Imaging the Reverse Bias Behavior of Perovskite Solar Cells

Jonathan Henzel Klaas Bakker Sjoerd Veenstra Olindo Isabella Luana Mazzarella Arthur Weeber Mirjam Theelen**

Jonathan Henzel
TNO, High Tech Campus 21, 5656 AE Eindhoven, The Netherlands
Delft University of Technology, Photovoltaic Materials and Devices, Mekelweg 4, 2628 CD Delft, Netherlands
Email Address: henzeljonathan@gmail.com

Dr. Klaas Bakker, Dr. Sjoerd Veenstra, Dr. Mirjam Theelen
TNO, High Tech Campus 21, 5656 AE Eindhoven, Netherlands
Email Address: mirjam.theelen@tno.nl

Prof. dr. Olindo Isabella, Dr. Luana Mazzarella, Prof. dr. Arthur Weeber
Delft University of Technology, Photovoltaic Materials and Devices, Mekelweg 4, 2628 CD Delft, Netherlands

SI Note 1: Fabrication process

In the following, we will describe the process of fabricating the perovskite solar cell that have been used in this study.

As substrates, we used 3 x 3 cm^2 soda-lime-glass substrate with a thickness of 1.1 mm. The bottom Indium-Tin-Oxide (ITO) electrodes ($\approx$ 150 nm) had already been deposited by full-area sputtering and photo-lithography. Our fabrication procedure begins with cleaning the substrates. To that end, we scrub the substrates (the top side, the edges, and the back side) in a soap solution. The substrates are sonicated for 10 min in the soap solution. After rinsing with deionized water, they are sonicated for 10 min in deionized water. Next, they are rinsed with isopropyl alcohol (IPA), and sonicated for 10 min in IPA. Finally, a UV/Ozone-cleaning step of 30 min follows. The substrates are introduced into a glovebox with nitrogen atmosphere where the following spin coating steps follow. We start by depositing the hole transport layer (HTL) ($\approx$ 10 nm) which is poly(triaryl amine) (PTAA) (Solaris Chem). To that end, we prepare a solution of PTAA in Toluene (2 mg/mL). This solution is spin coated dynamically on the cleaned substrates with 5000 rpm, 3000 $rpms^{-1}$ for 35 s. The solution is dropped about 5 s after the spinning started. Afterwards, all substrates are collected and annealed on a hotplate at 100° for 10 min. After a break of 15 min to allow for the atmosphere in the glovebox to recover, we proceed with the perovskite layer ($\approx$ 600 nm). The 1.4 M perovskite solution had been prepared one day prior. It is a triple-cation, double halide perovskite: $Cs_{0.05}FA_{0.8}MA_{0.15}PbI_{2.7}Br_{0.3}$, with Cs being cesium, FA formamidinium, MA methylammonium, Pb lead, I iodide, and Br bromide. This perovskite solution was created by solving PbI_2 (Thermo Fisher), $PbBr_2$ (TCI), $MABr_2$ (Dyenamo), FAI (greatcell solar), CsI (Thermo Fisher) in DMF/NMP (9:1). DMF is N,N-Dimethylformamide and NMP is Methylpyrrolidone. This solution was shortly before deposition heated up to 90° for 20 min and allowed to cool down over 1 h. It was dynamically spin coated on the substrates with first 2000 rpm, 200 $rpms^{-1}$, 10 s, then 5000 rpm, 2000 $rpms^{-1}$, 30 s by dropping the solution 5 s after starting the spinning. 20 s after starting the spinning, a nitrogen pistol was used for gas-quenching for 15 s. The substrates were immediately after deposition put onto a hotplate at 130° for 10 min. Afterwards, the electron transport layer (ETL) C_{60} ($\approx$ 20 nm) was deposited via thermal evaporation. Then, a buffer layer of SnO_2 ($\approx$ 50 nm) was deposited via spatial-ALD. Finally, the top electrode of ITO ($\approx$ 180 nm) was deposited via sputtering. The overlap area of the orthogonal bottom and top ITO electrodes is the cell area of 0.2 cm^2. The bottom electrode contacts were revealed by removing all layers except the bottom ITO, using tape and a DMF:chlorobenzene (1:6) solution.

SI Note 2: Results of the initial characterization

The initial characterization consisted of JV measurements on every cell and maximum-power-point-tracking on one cell from each substrate. The results of the JV measurements are shown in SI Figure 1.

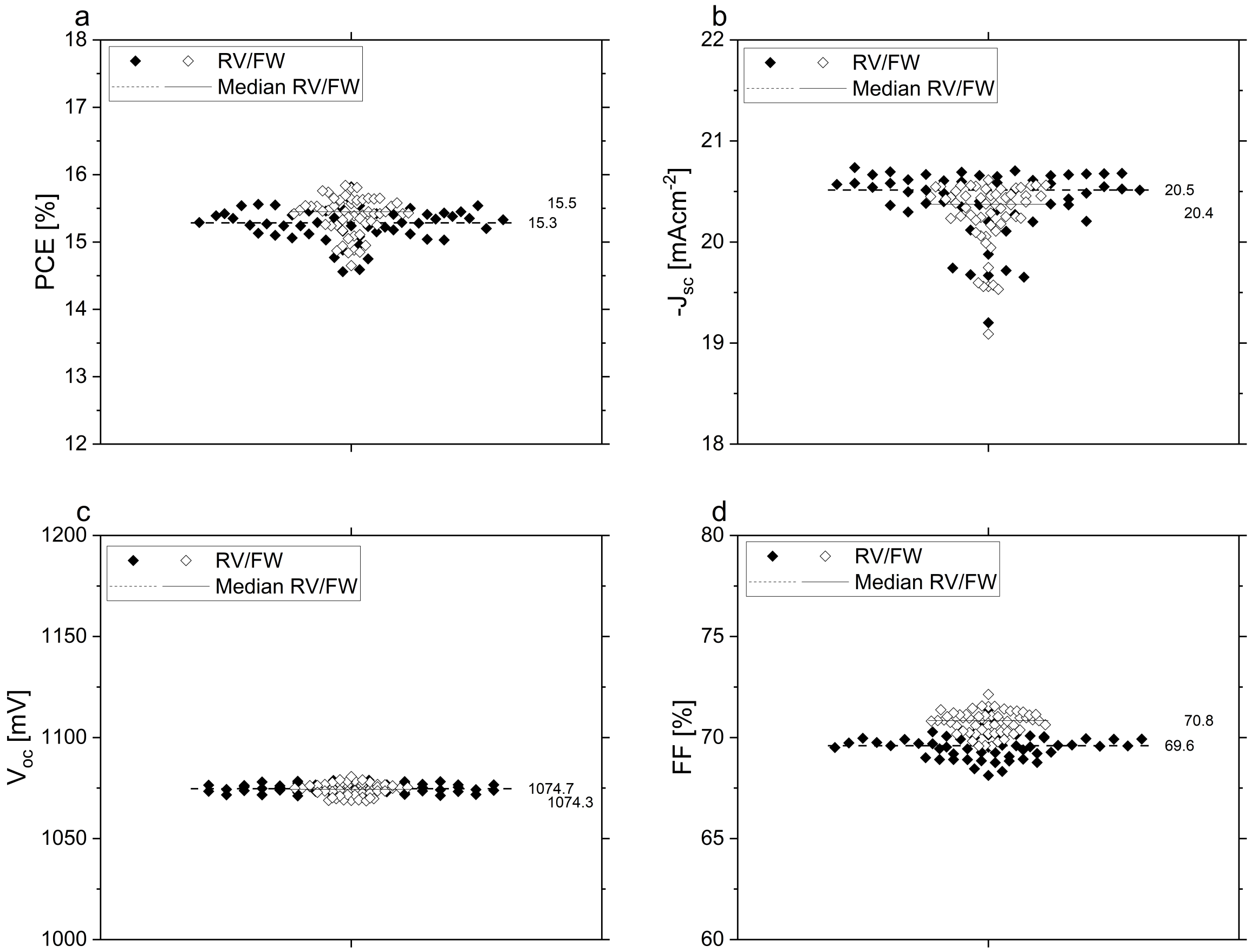

SI Figure 1: The results of JV measurements.

In SI Figure 2, the results of MPPT on one cell per substrate are displayed.

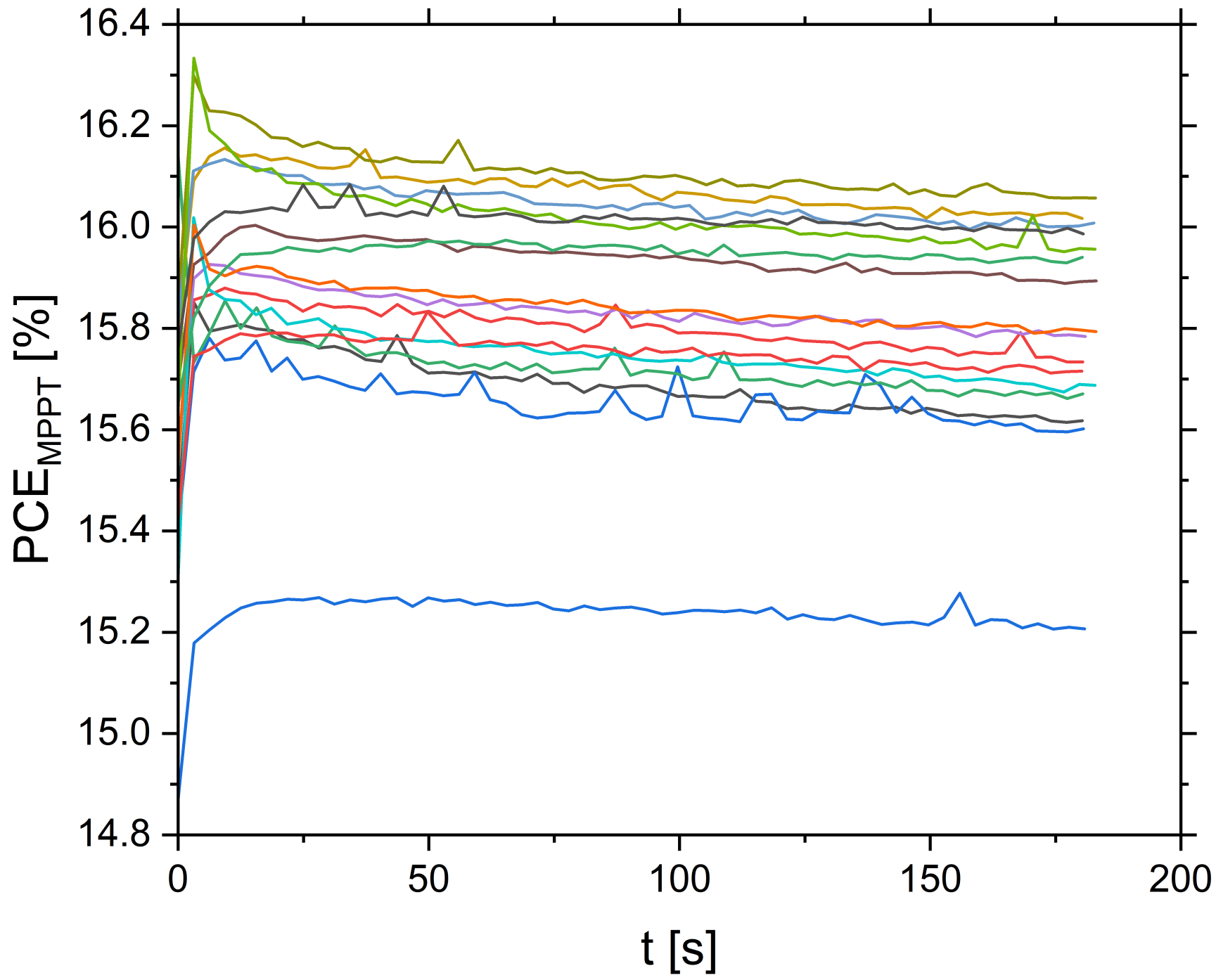

SI Figure 2: The results of MPPT.

For ReBEL measurements, we chose cells that showed good efficiencies and did not show any significant spots or other irregularities in electroluminescence. These images can be found in the Supplementary Data.

SI Note 3: Stabilizing the reverse bias behavior by repeated ReBEL imaging

In the experiment "ReBEL sweep", we repeated the ReBEL image-taking at every RB current level four times. For the analysis, we have only used each fourth image. The reason was that the reverse bias behavior of a perovskite solar cells needs time to stabilize. This is shown in Figure 3.

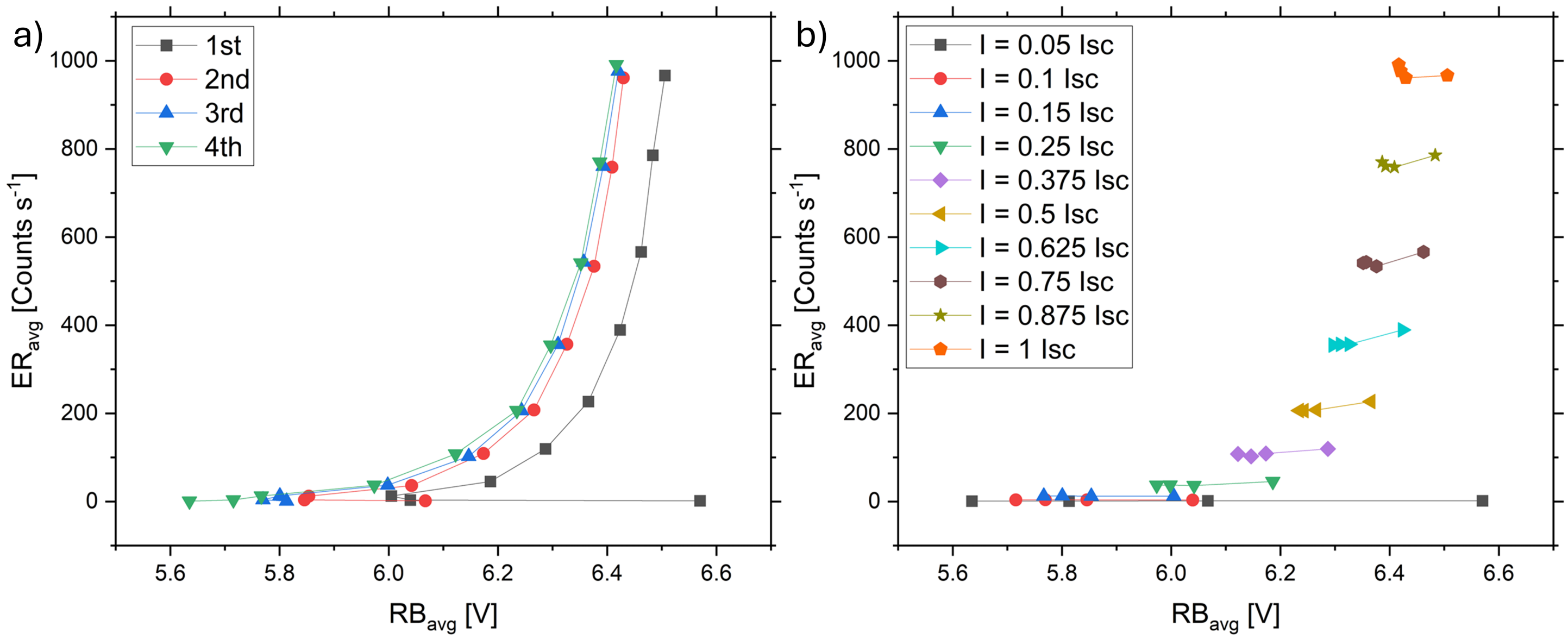

SI Figure 3: The impact of repeating the ReBEL image taking: The results of the quantitative analysis on a location in the cell area. In a), the data point are grouped (and colored) according to their affiliation to the first, second, third, or fourth repetition. In b), the same data points are grouped according to the RB current level.

There, we present the ER_{avg}-values of a small, square location in the cell area. They are plotted linearly against RB_{avg}. Instead of showing only the values from all fourth images, the complete data set is displayed. Figure 3a and b show the same data points; they are, however, sorted in different groups.

In Figure 3a, all the first, second, third, and fourth images are grouped together, respectively. We see that all four series show largely the same behavior, but with a voltage shift. A significant voltage difference is visible between the first and the second series. The second and third series are already closer together, and no difference is visible between the third and the fourth series.

Special attention should be devoted to the data points with very low emission rates. There, the differences between the RB_{avg} is largest. Especially one data point at about 6.6 V and its following data points stand out. Despite injecting the same current, the mean RB decreases from about 6.6 V to about 5.6 V. Only the fourth data point of that series fits in with the data points from larger currents. That is the reason why we compare all the fourth images and reverse biases in the main text.

In Figure 3b, the same data points are displayed, but in different groups. This time, they are sorted by RB current level. We see that the ER_{avg}-values within one group do not change significantly from the first to the fourth image. However, the RB_{avg} does change a lot. It is always significantly higher in the first image, and shifts during the following images to smaller RB_{avg}-values. We also see that especially the differences between the first and the second image is large. Only small additional changes occur between the second and the fourth image. Only at very small RB currents, these changes are even after four images still significant (see red circles, $I = 0.1 I_{sc}$).

This phenomenon is likely related to the slow movements of mobile ions towards the interfaces in the context of the breakdown mechanism. In order to correct for this meta-stable behavior, we have chosen to use only the fourth image of every RB current level. At the fourth repetition, the RB_{avg} seems to be stabilized for most RB current levels.

At the same time, these results reveal information on the influence of bias history within the ReBEL sweep. It is clearly visible that, whenever the current level is increased, the stabilization of mean reverse bias and emission rate begins anew. This implies that a measurement is not too strongly influenced by the respectively previous

measurement (at lower current level).

SI Note 4: Voltages during ReBEL measurement

During a ReBEL measurement, the voltage is not constant. For that reason, we calculate a mean voltage and use that in the following calculations as V. This is shown in SI Figure 4.

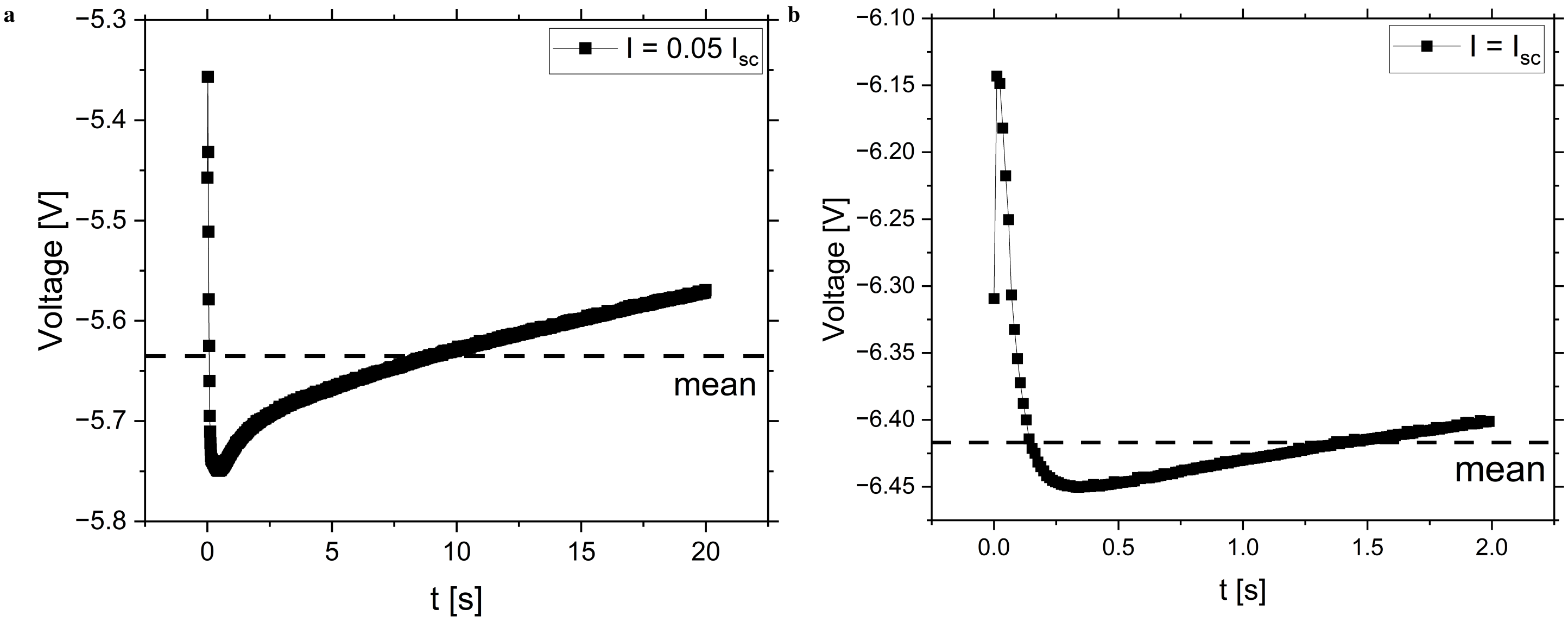

SI Figure 4: The voltages logged during ReBEL measurements at a small current (a) and at a large current (b) with the mean voltage that is called RB_{avg} in the following.

SI Note 5: Spectrally-dependent EL and ReBEL measurements

In SI Figure 5, the raw data, the baseline correction and the resulting spectra of the spectrally-dependent measurements are displayed.

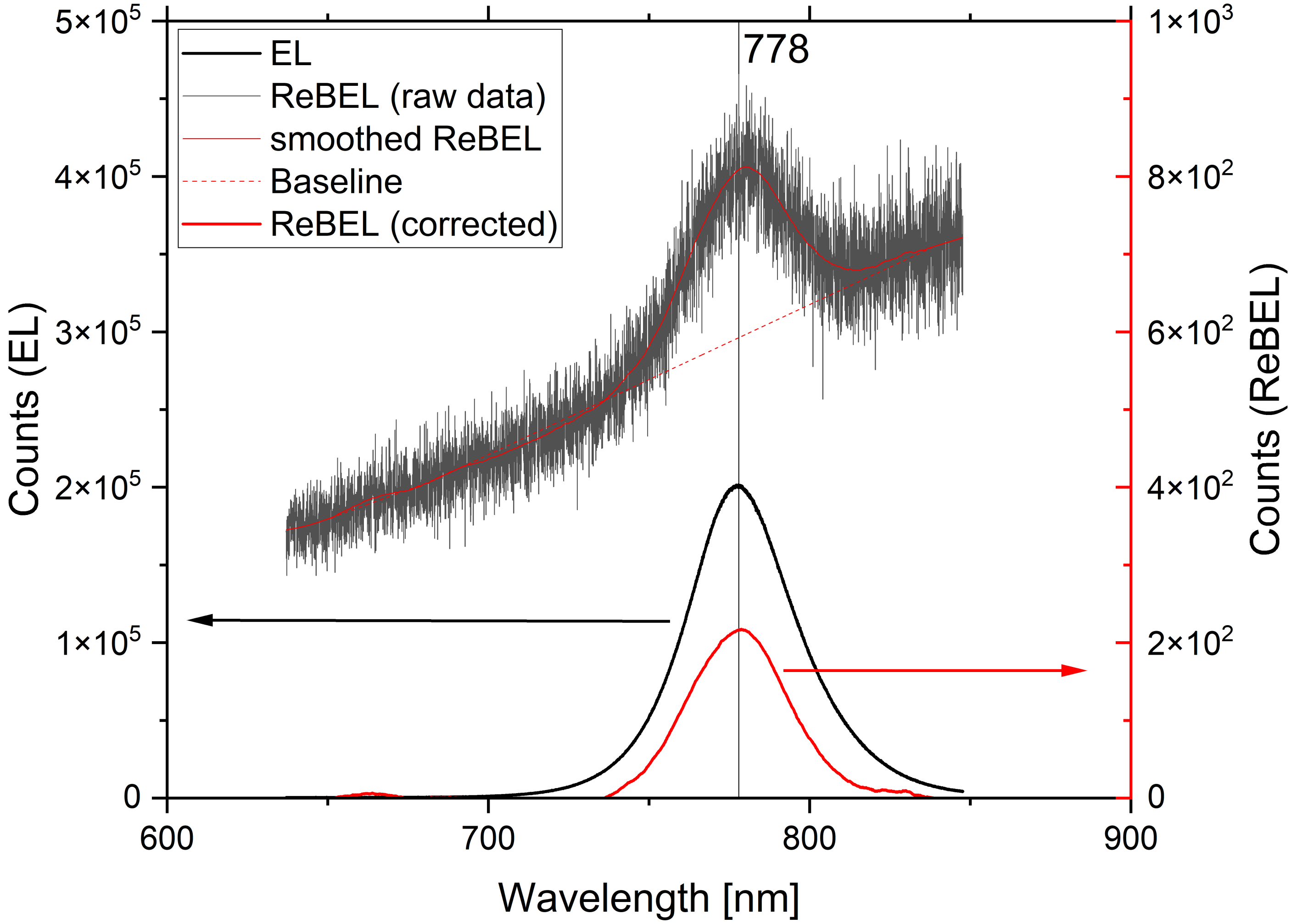

SI Figure 5: The EL (thick, black line) and ReBEL (thick, red line) spectrum. The raw data of the ReBEL spectrum is displayed as gray, thin line, the smoothed curve as red, thin, straight line. The baseline that is used for the baseline correction is the red, dashed line.

SI Note 6: Bright edges in ssIR images

Here, we show that the bright edges, that are visible in some ssIR images, are measurement artifacts. To that end, SI Figure 6 shows a series of ssIR images that are taken before the current injection started. The first image is used as baseline and therefore is completely dark. We observe that the edges are in some images barely visible, but in other images very bright or very dark. We conjecture that vibration of the camera, that is caused by the cooling Stirling motor, is responsible for these slight changes.

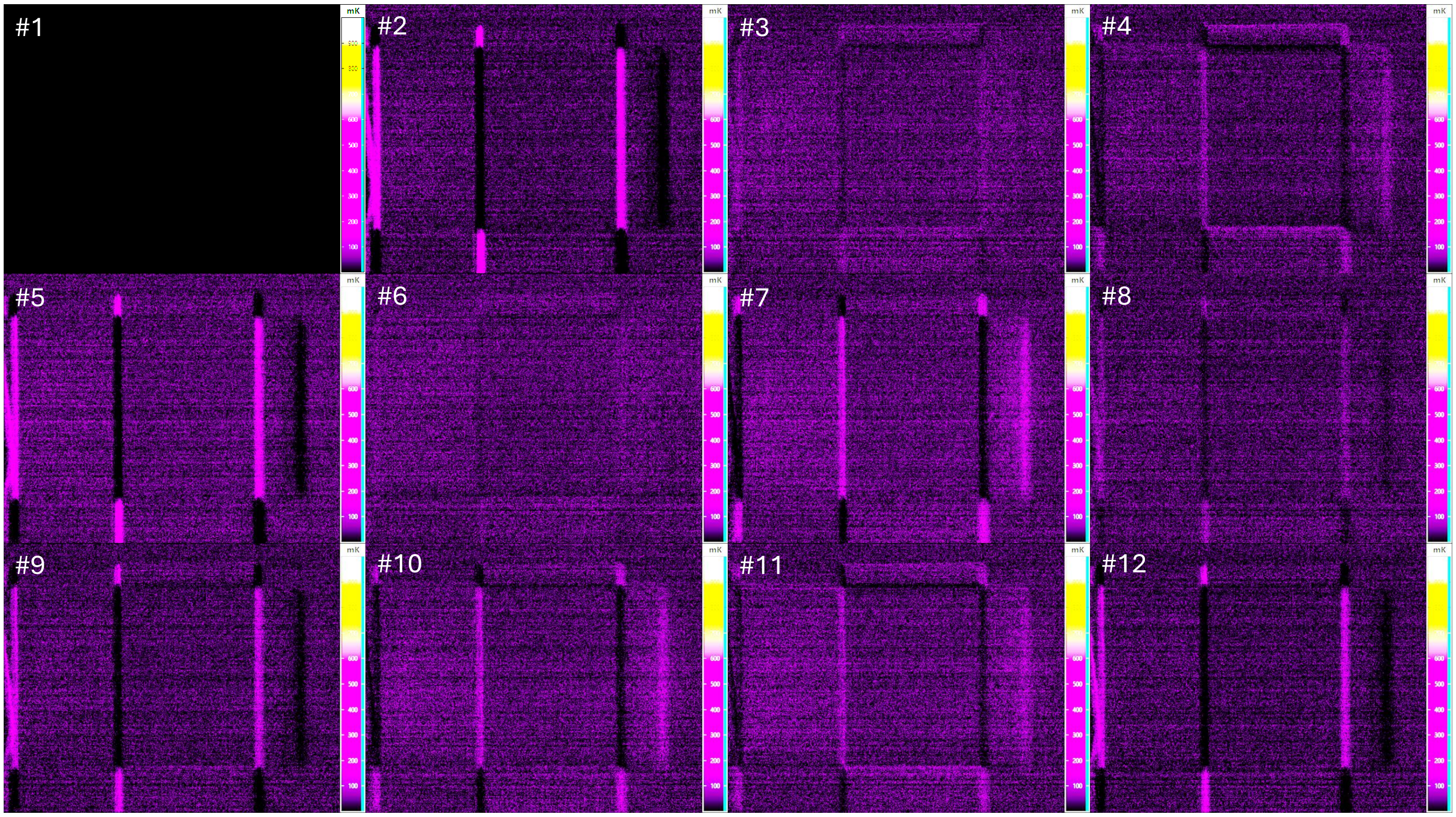

SI Figure 6: A series of twelve ssIR images, taken before the current injection started. To make the cell area visible at this size, the brightness of the images was increased.

SI Note 7: Additional semi-quantitative analysis

In the main text, we showed the semi-quantitative analysis of one example area each for the cell area, the bright edges, and the bright spots. Here, we give additional examples.
SI Figure 7 shows the semi-quantitative analysis of several small areas within the cell area. ReBEL images at a small and a large current show the locations of these small areas as blue squares. The graph in SI Figure 7c shows the average emission rates and reveals two regimes in the cell area: At the data points with a red background, the cell area has not yet begun lighting up. At voltages with a green background, the cell area has become active. All the points in the cell area behave qualitatively similar. The differences seem to amount largely to a offset in the emission rate.

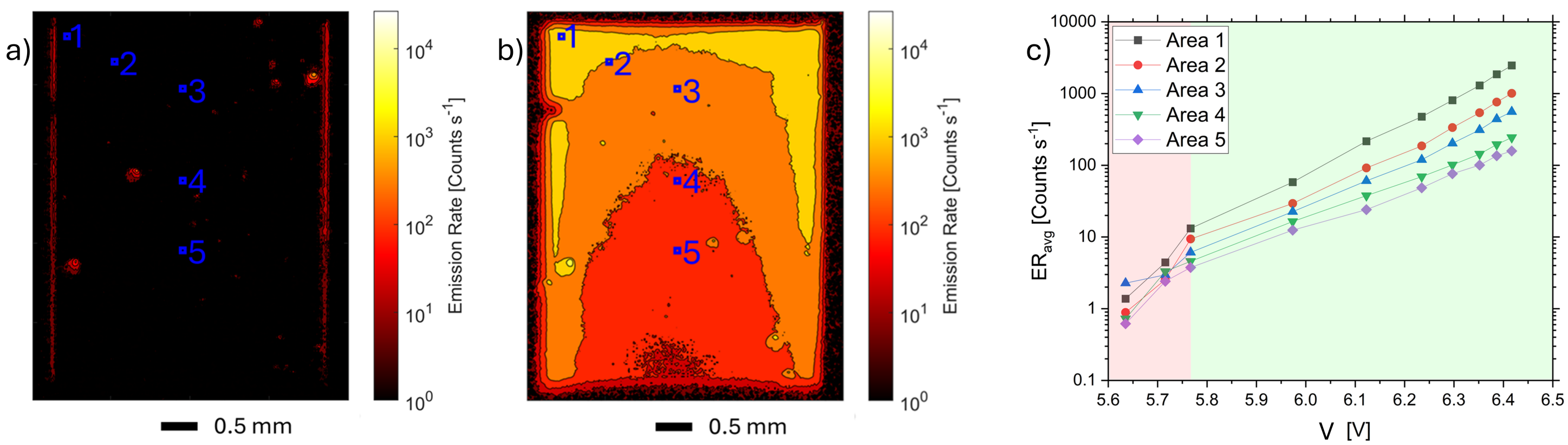

SI Figure 7: Five different points in the cell area are marked for semi-quantitative analysis in ReBEL images at a small current (a) and a large current (b). The average emission rates are plotted semi-logarithmically against the reverse bias (c). The background colors mark different regimes.

SI Figure 8 shows the semi-quantitative analysis of several different points along the cell edges. The positions of the investigated points are shown in ReBEL images at a small and a large current. Additionally, one point in the cell area is used as comparison. The graph displays the average emission rates semi-logarithmically plotted against the reverse bias. It clearly shows that all points at the edges behave the same way. At small voltages (red background), the edges are bright and turn brighter while the area is yet inactive. When the area begins to light up (green background), the emission rate of the edges saturates. At large reverse biases, the edges are not distinctly visible anymore in the ReBEL images. The emission rates at their former locations are 'pulled along' by the surrounding cell area.

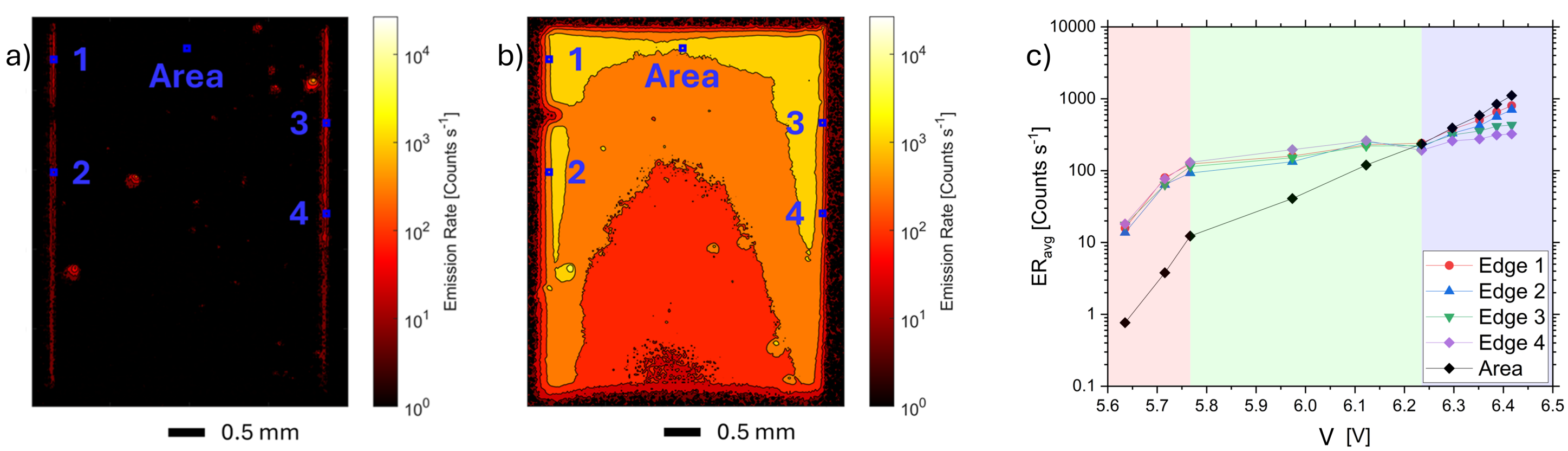

SI Figure 8: Four different points at the cell edges are marked for semi-quantitative analysis in ReBEL images at a small current (a) and a large current (b). A point in the cell area is added as comparison. The average emission rates are plotted semi-logarithmically against the reverse bias (c). The background colors mark different regimes.

SI Figure 9 shows the semi-quantitative analysis of several bright spots. Their location is shown in ReBEL images at a small and a large current. Additionally, one location in the cell area is used as comparison. The graph shows their average emission rates semi-logarithmically plotted against the reverse bias. It is clearly visible that several different behaviors are present among the bright spots. One commonality is, however, that their emission rates tend to saturate at large reverse biases.

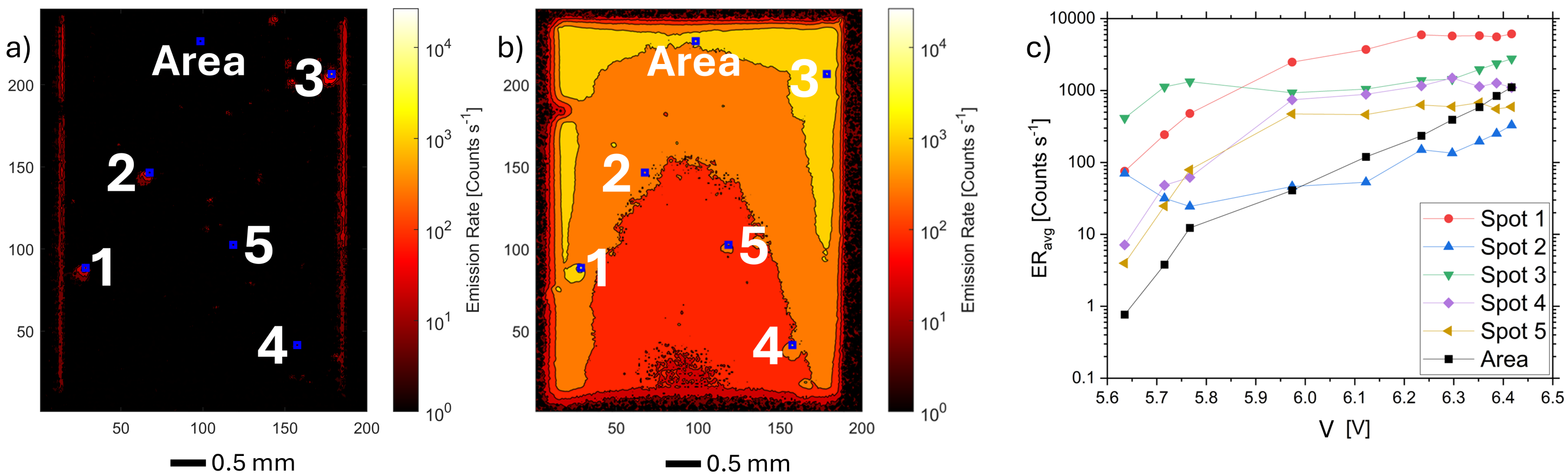

SI Figure 9: Five different bright spots are marked for semi-quantitative analysis in ReBEL images at a small current (a) and a large current (b). One point in the cell area is added as comparison. The average emission rates are plotted semi-logarithmically against the reverse bias (c).

SI Note 8: Profilometry results

Two different bottom ITO electrodes were tested in regards to their effect on the bright edges in ReBEL images. Profilometry was used to investigate the edge of the ITO stripes. SI Figure 10 shows the results. In SI Figure 10a, the edge of the standard ITO is shown, in SI Figure 10b, the edge of rounded ITO. We see that the standard ITO shows a very sharp, step-like edge while the rounded ITO displays a rounded edge. The green and red vertical bars were are markers for the calculation of the layer thickness.

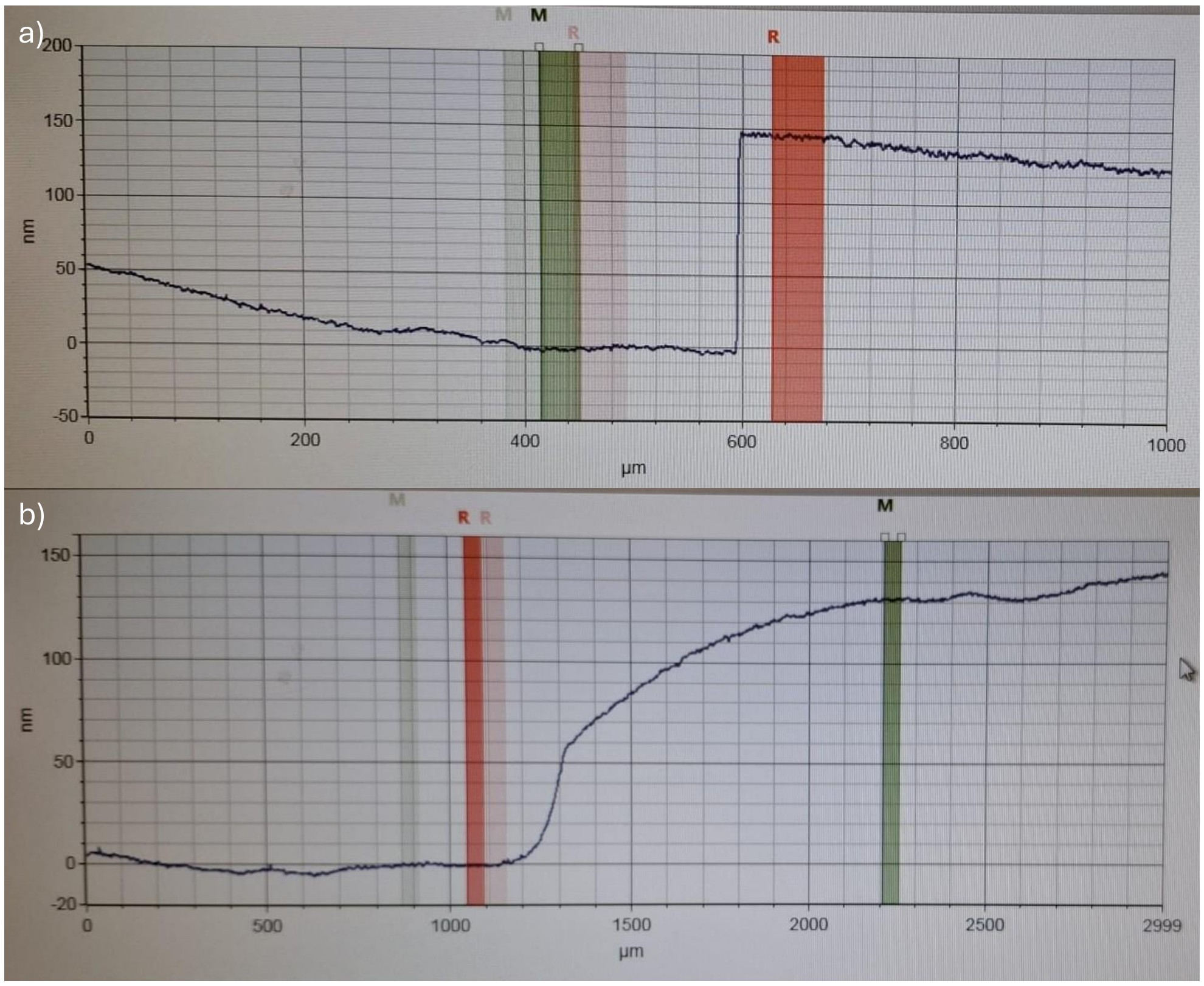

SI Figure 10: Results of profilometry measurements on standard (a) and self-sputtered ITO electrodes.
